# Regeneration of Vocal Fold Mucosa Using Tissue-Engineered Structures with Oral Mucosal Cells

**DOI:** 10.1371/journal.pone.0146151

**Published:** 2016-01-05

**Authors:** Mioko Fukahori, Shun-ichi Chitose, Kiminori Sato, Shintaro Sueyoshi, Takashi Kurita, Hirohito Umeno, Yu Monden, Ryoji Yamakawa

**Affiliations:** 1 Department of Otolaryngology-Head and Neck Surgery, Kurume University School of Medicine, Kurume, Fukuoka, Japan; 2 Department of Ophthalmology, Kurume University School of Medicine, Kurume, Fukuoka, Japan; Institute for Frontier Medical Sciences, Kyoto University, JAPAN

## Abstract

**Objectives:**

Scarred vocal folds result in irregular vibrations during phonation due to stiffness of the vocal fold mucosa. To date, a completely satisfactory corrective procedure has yet to be achieved. We hypothesize that a potential treatment option for this disease is to replace scarred vocal folds with organotypic mucosa. The purpose of this study is to regenerate vocal fold mucosa using a tissue-engineered structure with autologous oral mucosal cells.

**Study Design:**

Animal experiment using eight beagles (including three controls).

**Methods:**

A 3 mm by 3 mm specimen of canine oral mucosa was surgically excised and divided into epithelial and subepithelial tissues. Epithelial cells and fibroblasts were isolated and cultured separately. The proliferated epithelial cells were co-cultured on oriented collagen gels containing the proliferated fibroblasts for an additional two weeks. The organotypic cultured tissues were transplanted to the mucosa-deficient vocal folds. Two months after transplantation, vocal fold vibrations and morphological characteristics were observed.

**Results:**

A tissue-engineered vocal fold mucosa, consisting of stratified epithelium and lamina propria, was successfully fabricated to closely resemble the normal layered vocal fold mucosa. Laryngeal stroboscopy revealed regular but slightly small mucosal waves at the transplanted site. Immunohistochemically, stratified epithelium expressed cytokeratin, and the distributed cells in the lamina propria expressed vimentin. Elastic Van Gieson staining revealed a decreased number of elastic fibers in the lamina propria of the transplanted site.

**Conclusion:**

The fabricated mucosa with autologous oral mucosal cells successfully restored the vocal fold mucosa. This reconstruction technique could offer substantial clinical advantages for treating intractable diseases such as scarring of the vocal folds.

## Introduction

The human vocal fold has a layered structure that consists of the epithelium, lamina propria (superficial, intermediate, and deep layers), and vocalis muscle. The epithelium and lamina propria are especially important for vocal fold vibrations during phonation. Vocal fold scarring can be caused by surgery or iatrogenic injury to the layered structure of the vocal folds. It can lead to replacement of healthy tissue in the vocal fold lamina propria by fibrous tissue, and can disrupt the stratification of the vocal fold epithelium [1, 2].

Scarring on the vocal folds creates irregular vibrations due to viscoelastic changes in the vocal fold mucosa, and leads to severe hoarseness during communication. The management of this condition is challenging for otolaryngologists because effective therapy is currently lacking. To date, several approaches have been used to address scarring of the vocal folds, including medialization laryngoplasty procedures [3–6], mucosa grafting [7], the use of angiolytic lasers [8], and tissue engineering [9–11] involving cell delivery, growth factors, and pharmacological agents. The principle of tissue engineering holds that the introduction of a fibroblast population into the scarred tissue of the vocal folds could theoretically lead to reconstitution of lamina propria components and reestablishment of normal mucosal waves.

There has been limited discussion regarding the importance of the epithelium of the vocal fold mucosa. Optimal mucosal vibrations depend not only on the proper viscoelasticity of the superficial layer of the lamina propria, but also on the uniformity of the stratified epithelium in the vocal fold mucosa. A potential treatment option for scarred vocal folds is to replace the vocal fold mucosa. Rapid re-epithelialization is required, especially when the scarred vocal fold is resected, to create a physical barrier that protects the compromised tissue in the acute phase of wound healing.

The aim of this study is to develop a regeneration technique for the vocal fold mucosa using a tissue-engineered structure containing autologous oral mucosal cells.

## Materials and Methods

### Experimental animals

All experimental protocols used in this study were approved by the Kurume University Animal Care and Treatment Committee. Eight female beagles (KBT Oriental, Saga, Japan) weighing 11.3 to 13.8 kg were caged individually with free access to standard laboratory chow and tap water. Individual cage sizes were 950 millimeters (width) x 1000 millimeters (depth) x 1920 millimeters (height). The fabrication of tissue-engineered vocal fold mucosa was conducted using the procedures described below.

### Cell harvesting, isolation, and culture

Experimental animals were anesthetized by intramuscular administration of xylazine (2 mg/kg) and midazolam (0.3 mg/kg), and with intramuscular administration of ketamine (5 mg/kg). An endotracheal tube was inserted and general anesthesia was maintained by intravenous administration of pentobarbital sodium (10-20mg/kg).

**Fig 1:**
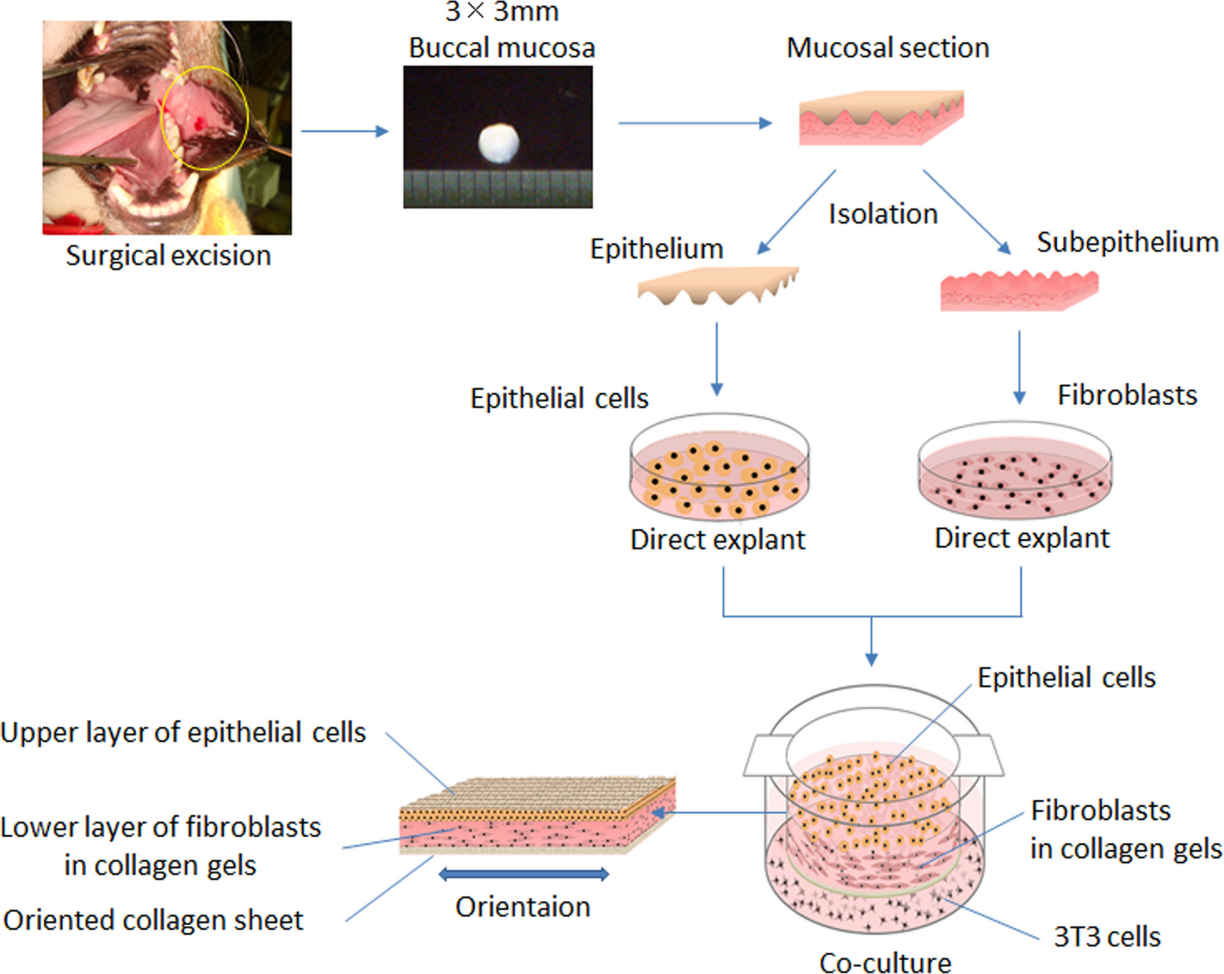
Fabrication protocol of tissue-engineered vocal fold mucosa. A 3 mm by 3 mm specimen of buccal mucosal tissue was excised. The mucosal section was divided into the epithelium and subepithelium. Isolated epithelial cells from the epithelium and isolated fibroblasts from the subepithelium were separately cultured for a two-week culture period. Following this, using cell-culture inserts, the proliferated fibroblasts were mixed into concentrated type I collagen gel on an oriented collagen sheet and cultured for one week. The proliferated epithelial cells were seeded and co-cultured on the collagen gel containing fibroblasts. After an additional two-week co-culture period, fabrication of the organotypic cultured tissue with stratified epithelial cells was complete.

A 3 mm by 3 mm specimen of buccal mucosal tissue was surgically excised from each of the five dogs (Fig 1). The specimen, excluding muscle, was washed with Dulbecco’s phosphate-buffered saline (PBS) containing antibiotics, and divided into mucosal and submucosal sections using a sterile scalpel under a stereoscopic microscope. Mucosal sections were incubated in Dulbecco’s Modified Eagle’s Medium/Nutrient Mixture F-12 (DMEM/F12, Gibco) containing 1.2 U/mL of dispase II (Roche), at 37°C for one hour. A thin epithelial layer was then peeled from the subepithelium using two sterile forceps under a stereoscopic microscope. Collected materials were placed in trypsin and ethylenediaminetetraacetic acid (EDTA) for seven minutes at 37°C to form single-cell suspensions. Approximately 5 x 10^5^ suspended epithelial cells were obtained per epithelial layer.

The following culturing technique for epithelial cells was modified based on previously reported methods [12, 13]. Epithelial cells were then cultured in a keratinocyte culture medium composed of DMEM/F-12, supplemented with 10 ng/mL epidermal growth factor (Sino Biological), 10 μg/mL insulin (Wako), 0.5 μg/mL hydrocortisone (Wako), 0.25 μg/mL isoproterenol (Wako), 1.3 ng/mL triiodothyronine (MP Biomedicals), 100 units/mL penicillin, 100 μg/mL streptomycin, 0.25 μg/mL amphotericin B, and 4% fetal bovine serum (FBS, Gibco) throughout a two-week culture period.

During this time, fibroblasts from the subepithelium were also isolated and cultured separately. The submucosal sections were minced into smaller particles, measuring 1 mm^2^, in a minimal amount of Hank’s Balanced Salt Solution (Invitrogen). Submucosal particles were cultured for cell migration on a 35-mm dish (Falcon) containing DMEM/F-12 and 10% FBS throughout a two-week culture period.

Following this, confluent epithelial cells and fibroblasts were obtained as single cell suspensions by treatment with trypsin and EDTA for seven minutes each.

### Fabrication of the organotypic cultured vocal fold mucosa

First, using cell-culture inserts (Falcon), the isolated fibroblasts were mixed into type I collagen gel (Koken) containing 10X concentrated DMEM/F-12 and 10% FBS on an oriented collagen sheet (Atree) and cultured at 37°C in a humidified atmosphere of 5% carbon dioxide for one week.

The proliferated epithelial cells were seeded and co-cultured on shrinkage collagen gel containing fibroblasts at a density of 8 x 10^4^ /cm^2^ and were incubated with a mitomycin C-treated 3T3 feeder layer at 37°C in a humidified atmosphere of 5% carbon dioxide. After an additional two-week co-culture period, the fabrication of the organotypic cultured tissue with stratified epithelial cells was complete.

### Evaluation of the organotypic cultured vocal fold mucosa

After the fabricated tissues were harvested, their morphological characteristics were observed. Observations of the surfaces of the organotypic cultured vocal fold mucosa were conducted using a Hitachi S-800 scanning electron microscope. Frozen sections from the other fabricated tissues were stained with Hematoxylin and Eosin (H&E). Immunohistochemical staining was performed with a polyclonal antibody against cytokeratin 8 intermediate filament protein (MyBioSource, San Diego, CA) and a monoclonal antibody against vimentin (Abcam plc, Cambridge, UK). Cytokeratin 8 is expressed in normal oral epithelial cells, but not in vocal fold epithelial cells [14, 15]. Vimentin, an intermediate filament protein, is expressed in a large variety of cells, including fibroblasts, endothelial cells, macrophages, neutrophils, and lymphocytes, and serves as an indicator of cell proliferation in wound healing [16]. Immunoreactivity was examined by light microscopy (Nikon). The same concentration of corresponding normal non-specific IgG was used for negative controls, and normal canine oral mucosa was used as the positive control.

### Transplantation to the mucosa-deficient vocal fold

**Fig 2:**
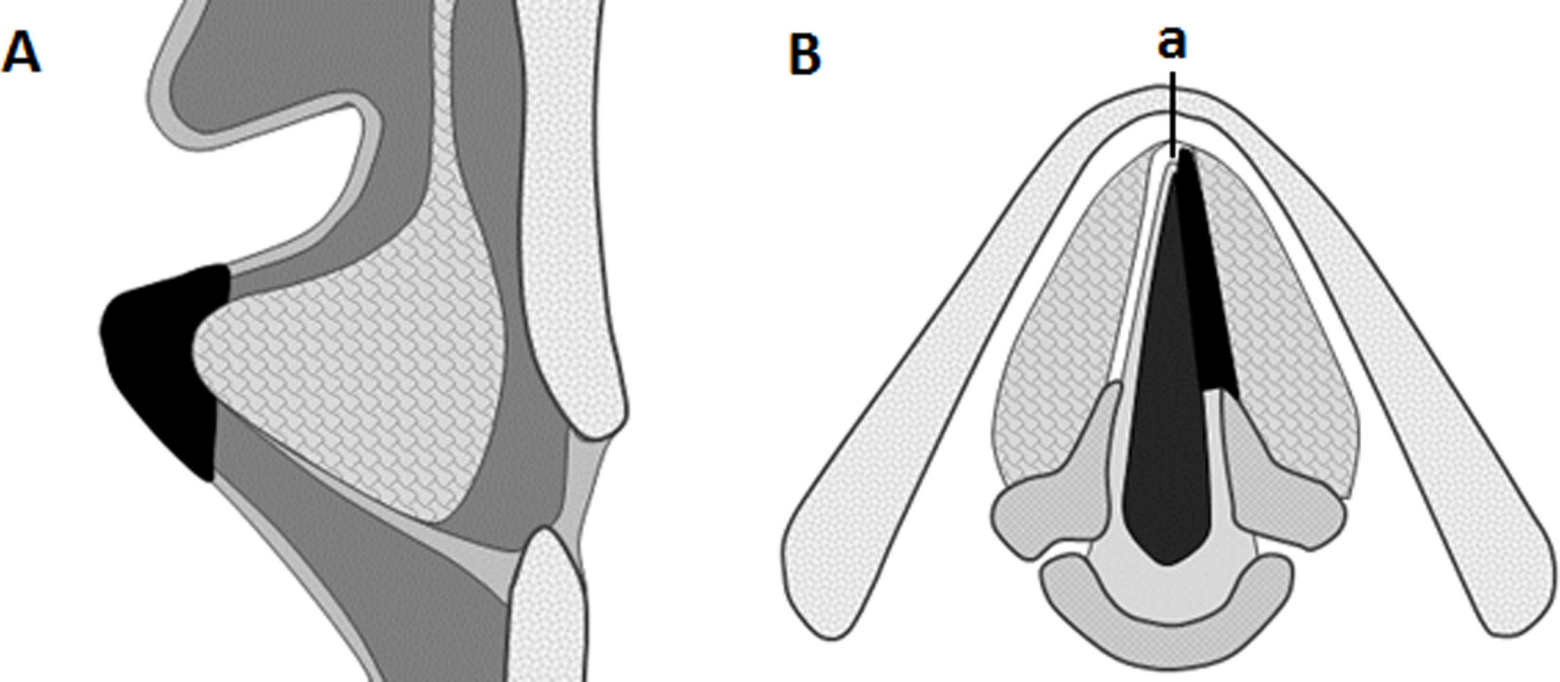
Schematic view of the extent of vocal fold resection. Vocal fold mucosa was resected to the full depth of the lamina propria in the coronal view (A). The vocal muscle was preserved to the extent possible. The superior to inferior dimensions of injury were about one-third the thickness of the vocal fold. The resection extended from the vocal process to the anterior commissure in the horizontal view (B). Line a: median approach by laryngofissure.

After laryngofissure was performed under general anesthesia with endotracheal intubation, the unilateral membranous portion of the vocal fold was resected according to the cordectomy type II method proposed by the European Laryngological Society in 2000 [17] (Fig 2A and 2B). Cordectomy was performed by cutting between the vocal mucosa and vocalis muscle, i.e., resection of the full depth of the lamina propria. The vocalis muscle was preserved to the extent possible. The resection extended from the vocal process to the anterior commissure. The superior to inferior dimensions of the resection were about one-third of the vocal fold thickness. In the five dogs for which mucosae were harvested, the resected area was immediately covered with tissue-engineered vocal fold mucosa using fibrin glue and fixed with absorbable surgical sutures (7–0 Vicryl, Ethicon) in four locations (anterior, posterior, superior, and inferior) under an operating microscope. During this time, the fabricated organotypic cultured tissue was transplanted along the same direction as the vocal fold. The antibiotics cefazolin sodium hydrate (20 mg/kg/day; for prevention of infection) and acetaminophen (10 mg/kg; analgesic) were administered after surgery.

### Evaluation of removed larynges

**Fig 3:**
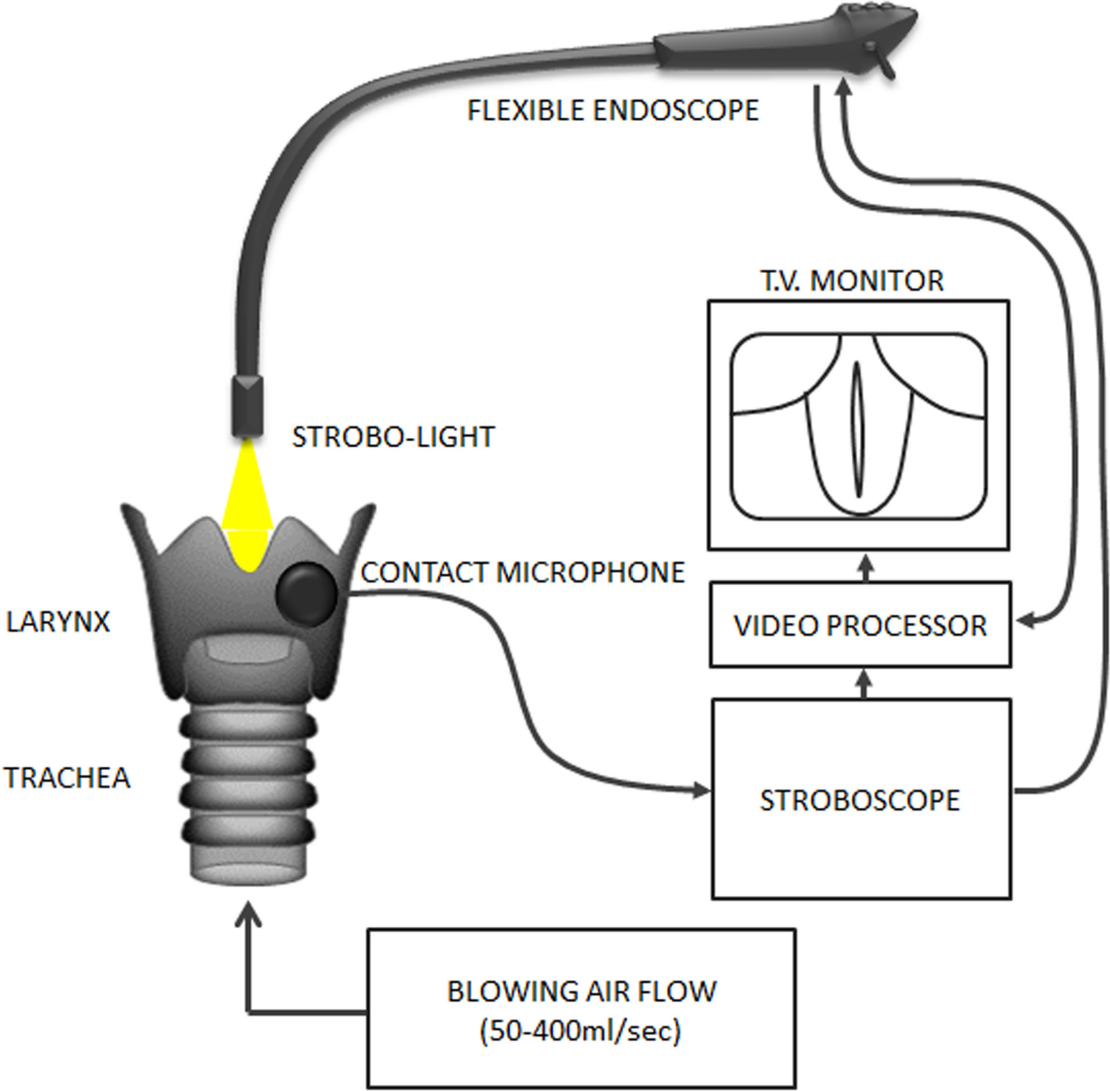
Diagram of stroboscopic observation of removed larynx. Glottal closure was achieved at the posterior portion by suturing the bilateral cartilaginous portion of the vocal folds of the excised larynges. A contact microphone was attached firmly to the excised larynges and connected to the laryngostroboscope. Experimental phonation was artificially induced by blowing air through the trachea. The upper surfaces of the vocal folds were illuminated by the stroboscopic light from the laryngostroboscope via a flexible endoscope. Vocal fold vibrations were recorded by a video processor.

Two months after transplantation, the five experimental animals and three controls were euthanized by intravenous injection of pentobarbitone solution (100 mg/kg), and the larynges were removed. Vocal fold vibrations caused by blowing air through the trachea were observed using laryngostroboscopy (Fig 3). Glottal closure was achieved by suturing the bilateral cartilaginous portion of the vocal folds of the removed canine larynges. A contact microphone was attached firmly to the excised larynges and connected to the laryngostroboscope (LS-3A, Nagashima). Experimental phonation was artificially induced by blowing air (50–400 ml/sec) through the trachea. The upper surfaces of the vocal folds were illuminated by a stroboscopic light from the laryngostroboscope though a flexible endoscope (ENF-VH, Olympus). Vocal fold vibrations were recorded by a video processor (OTV-S190, Olympus).

The removed larynges were then fixed in 4% paraformaldehyde. Coronal whole laryngeal sections were made at a thickness of 10 μm. At the middle of the membranous portion of the vocal fold, histological examinations were performed using H&E, Elastic Van Gieson (EVG), and immunohistochemical staining with anti-cytokeratin and anti-vimentin antibodies.

## Results

### Fabrication and evaluation of organotypic cultured vocal fold mucosa

**Fig 4:**
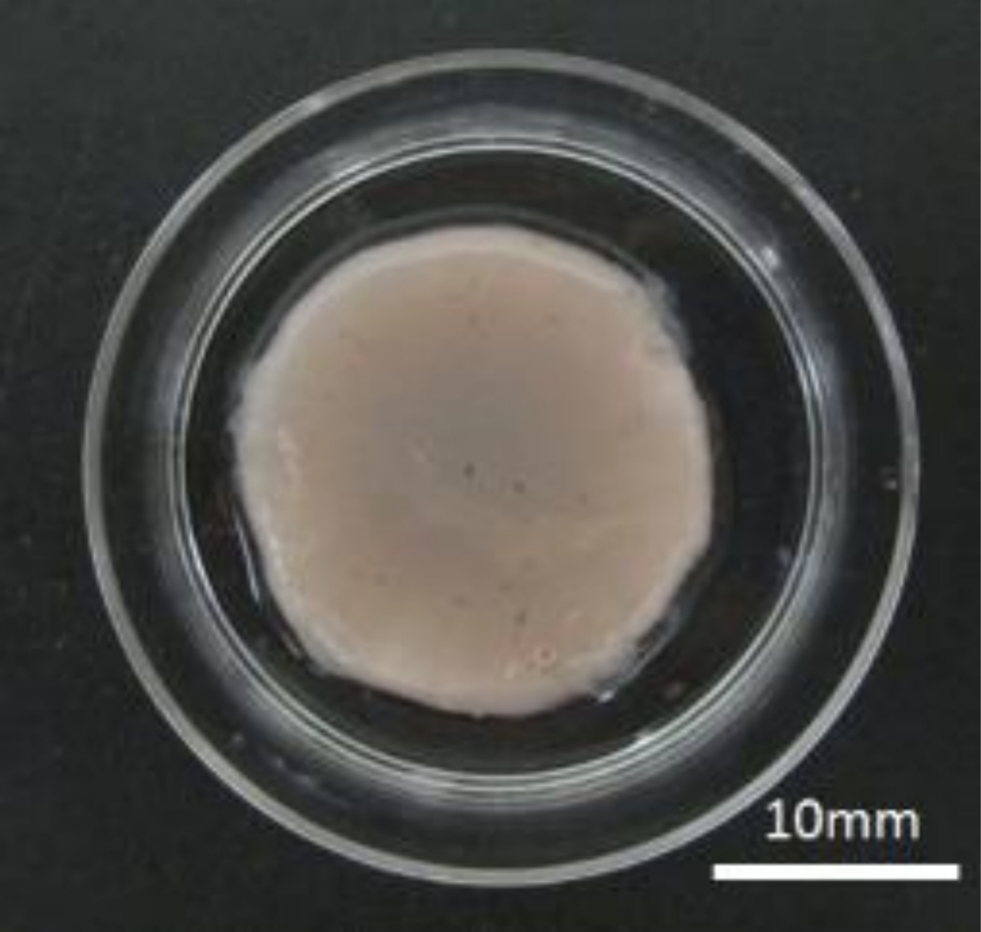
Tissue-engineered vocal fold mucosa *in vitro*. A tissue-engineered vocal fold mucosa was successfully fabricated (20 mm diameter).

**Fig 5:**
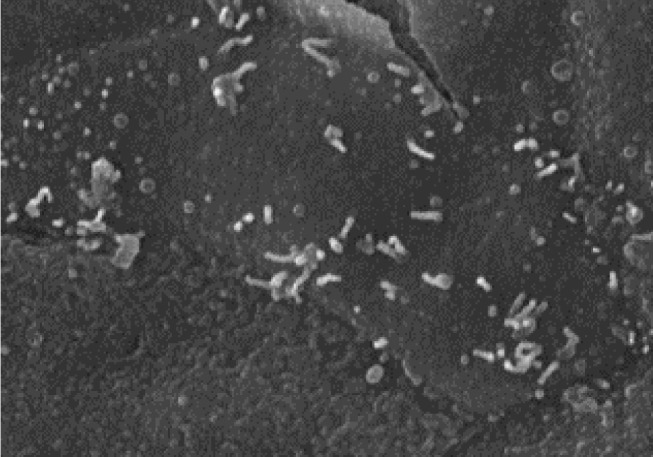
Scanning electron microscopy of tissue-engineered vocal fold mucosa. Microvilli developed on the epithelium of the tissue.

**Fig 6:**
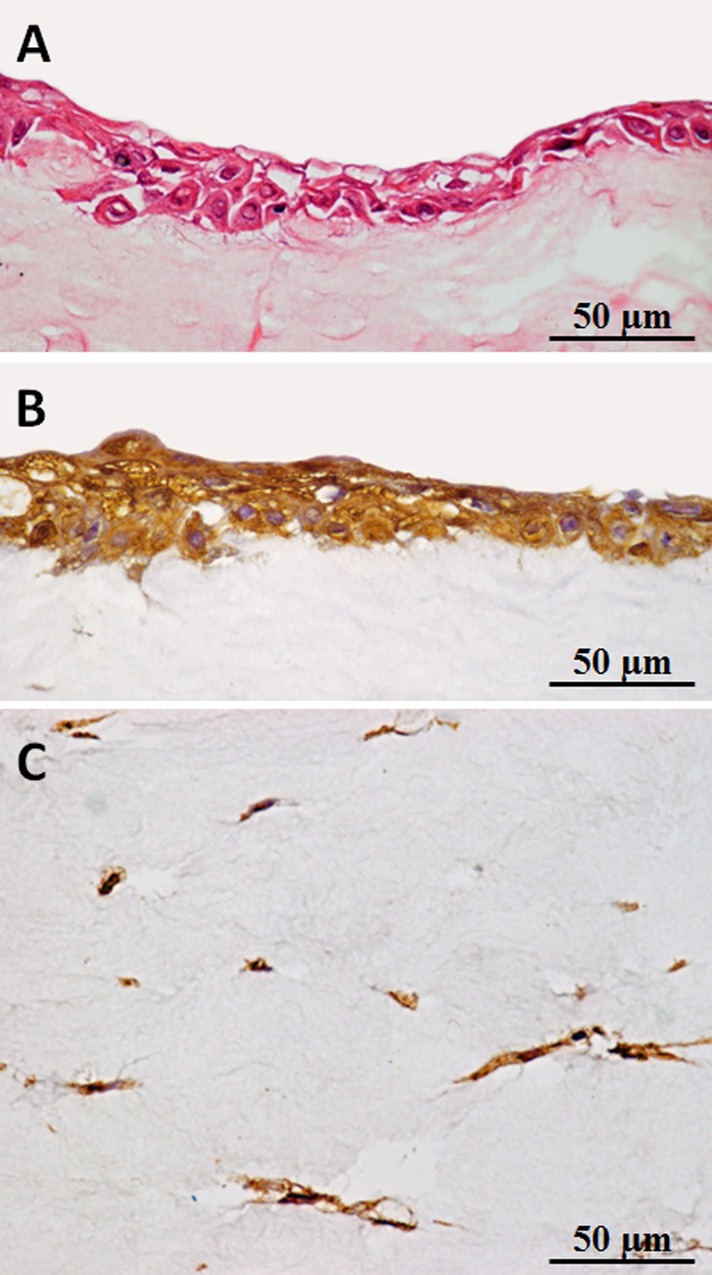
Evaluation of an organotypic cultured vocal fold. Hematoxylin and Eosin staining revealed that the tissue-engineered vocal fold mucosa consisted of the epithelium and lamina propria. The epithelium comprised two to four cell layers (A). Cells in the upper layer of the tissue-engineered vocal fold mucosa immunohistochemically expressed cytokeratin (B). Cells in the lower layer of the tissue-engineered vocal fold mucosa immunohistochemically expressed vimentin (C).

A tissue-engineered vocal fold mucosa was successfully fabricated *in vitro* (Fig 4). Scanning electron microscopy revealed that microvilli had developed on the epithelium of the tissue (Fig 5), and H&E staining showed the tissue-engineered vocal fold mucosa closely resembled normal vocal folds, consisting of epithelium and lamina propria. The stratified epithelium consisted of two to four cell layers (Fig 6A), and differed from the normal oral mucosa. Epithelial cells of the tissue-engineered vocal fold mucosa immunohistochemically expressed cytokeratin (Fig 6B), a keratin-containing intermediate filament found in the intracytoplasmic cytoskeleton of the epithelium. Cells in the tissue-engineered vocal fold lamina propria immunohistochemically expressed vimentin (Fig 6C). Almost all cells in the lamina propria were identified as fibroblasts.

### Transplantation to the mucosa-deficient vocal fold

**Fig 7:**
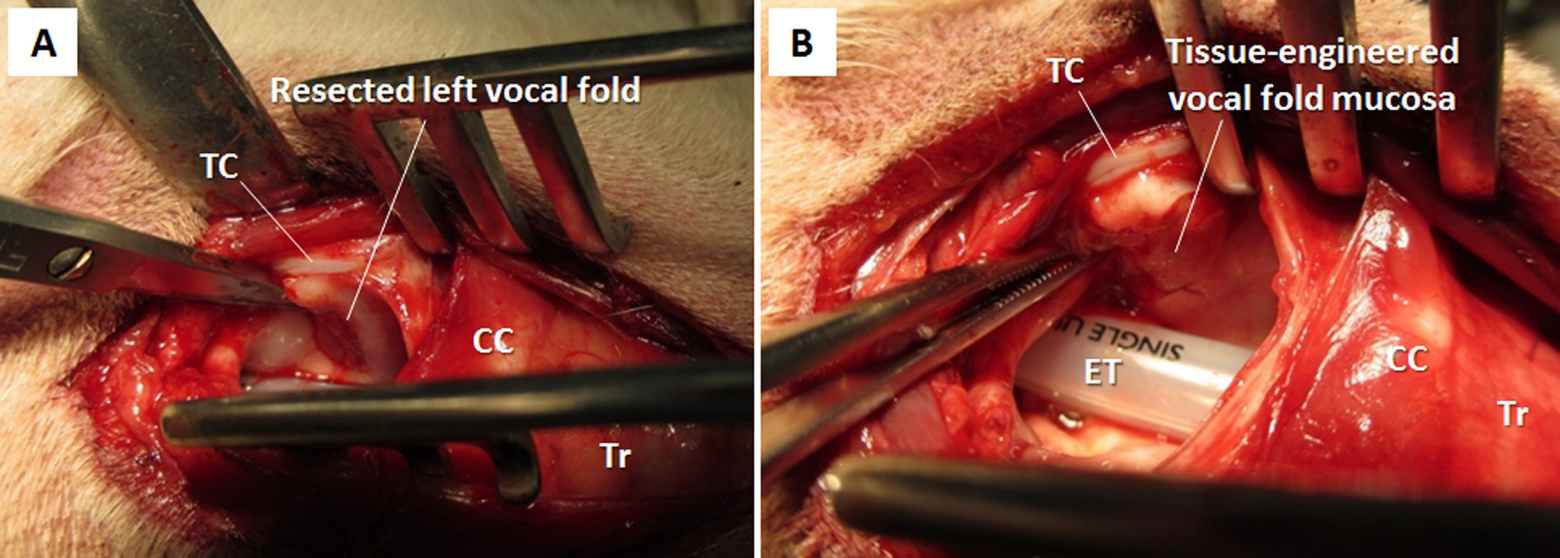
Transplantation of an organotypic cultured vocal fold to the mucosa-deficient vocal fold. After laryngofissure was performed, the unilateral membranous portion of the vocal fold was resected (A) to the extent shown in **Fig 2**. The organotypic cultured vocal fold was transplanted to the mucosa-deficient vocal fold (B). TC: median cut surface of thyroid cartilage, CC: cricoid cartilage, Tr: trachea, ET: endotracheal tube.

The tissue-engineered vocal fold mucosa was successfully transplanted onto the resected portion of the vocal folds (Fig 7A and 7B). These grafts were observed after transplantation every week using an Airway Mobilescope (MAF, Olympus) under general anesthesia without endotracheal intubation. During the postoperative observation period, transplanted tissues survived and integrated with the surrounding tissue. There was no clear delineation between the transplanted tissue and surrounding tissue two months after transplantation.

### Evaluation of removed larynges

**Fig 8:**
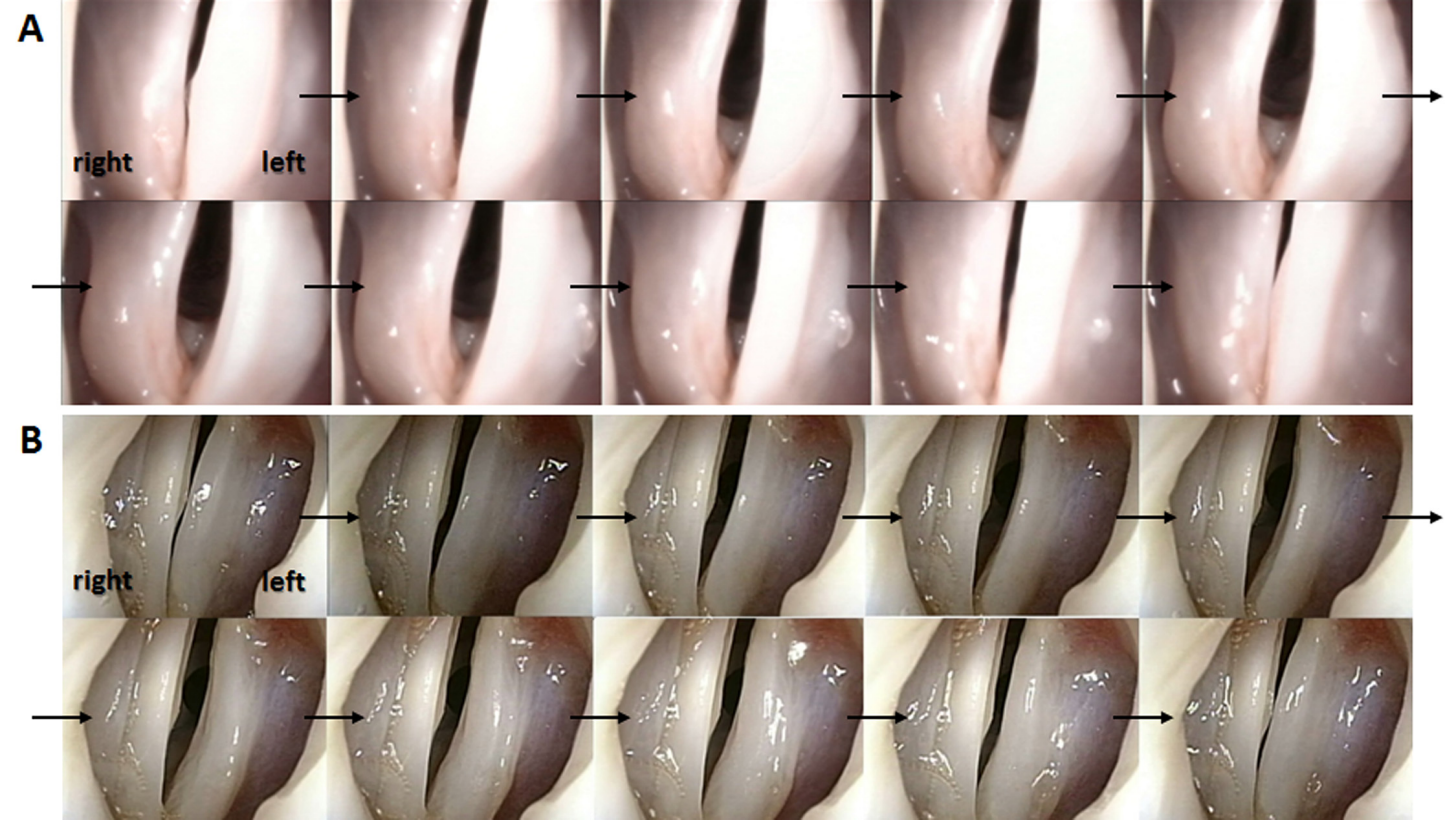
Stroboscopic findings. Mucosal waves on the transplanted side (right vocal fold) were regular but slightly smaller than those on the normal side (left vocal fold) (A). Mucosal waves on the control side (right vocal fold) in the controls were few (B).

Laryngostroboscopic examination revealed that mucosal waves on the transplanted side of the five canine larynges were regular but slightly smaller than those on the normal side (Fig 8A). Mucosal waves on the resected side in the three controls were few, whereas they were regular on the normal side (Fig 8B).

**Fig 9:**
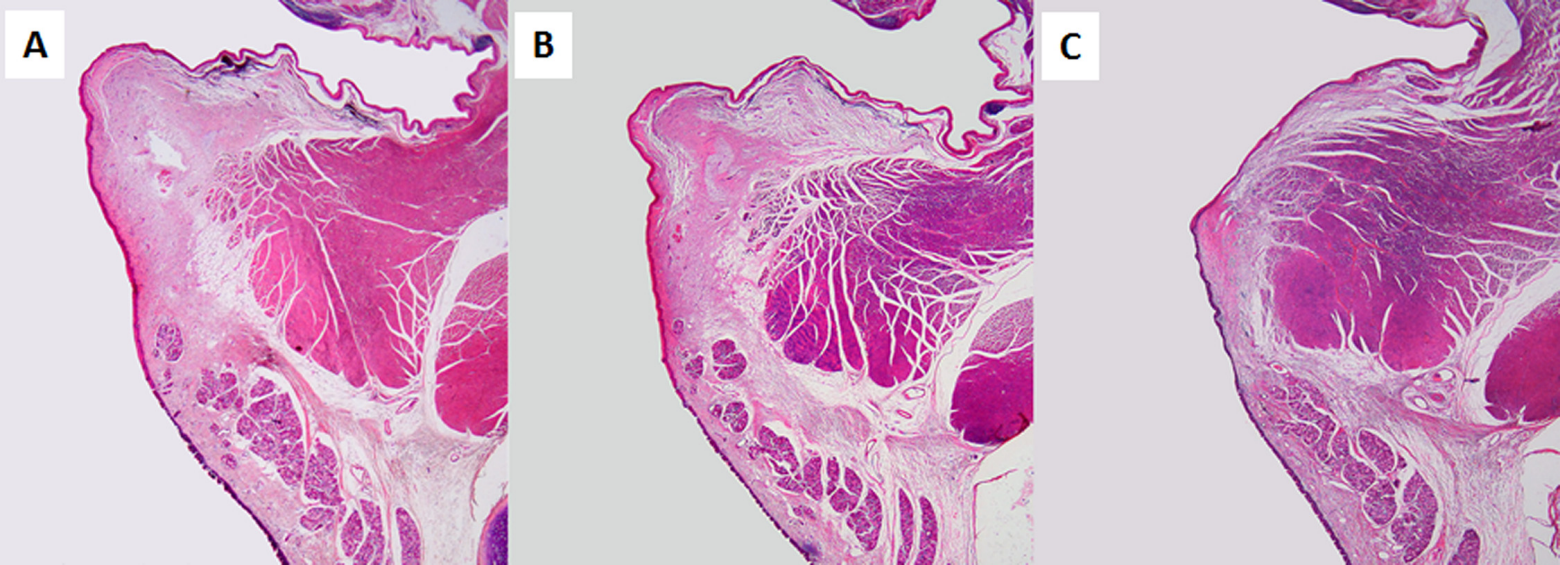
Whole organ section of a removed larynx (H&E stain). The regenerated vocal fold mucosa on the transplanted portion (B) was morphologically similar to that of the normal side (A). The vocal fold mucosa on the control side (C) was morphologically atrophic (B).

Whole laryngeal sections stained with H&E revealed that the regenerated vocal fold mucosae on the transplanted portion (Fig 9B) were morphologically similar to those on the normal vocal fold mucosae (Fig 9A). Vocal fold mucosae on the resected portion in controls (Fig 9C) were morphologically atrophic.

**Fig 10:**
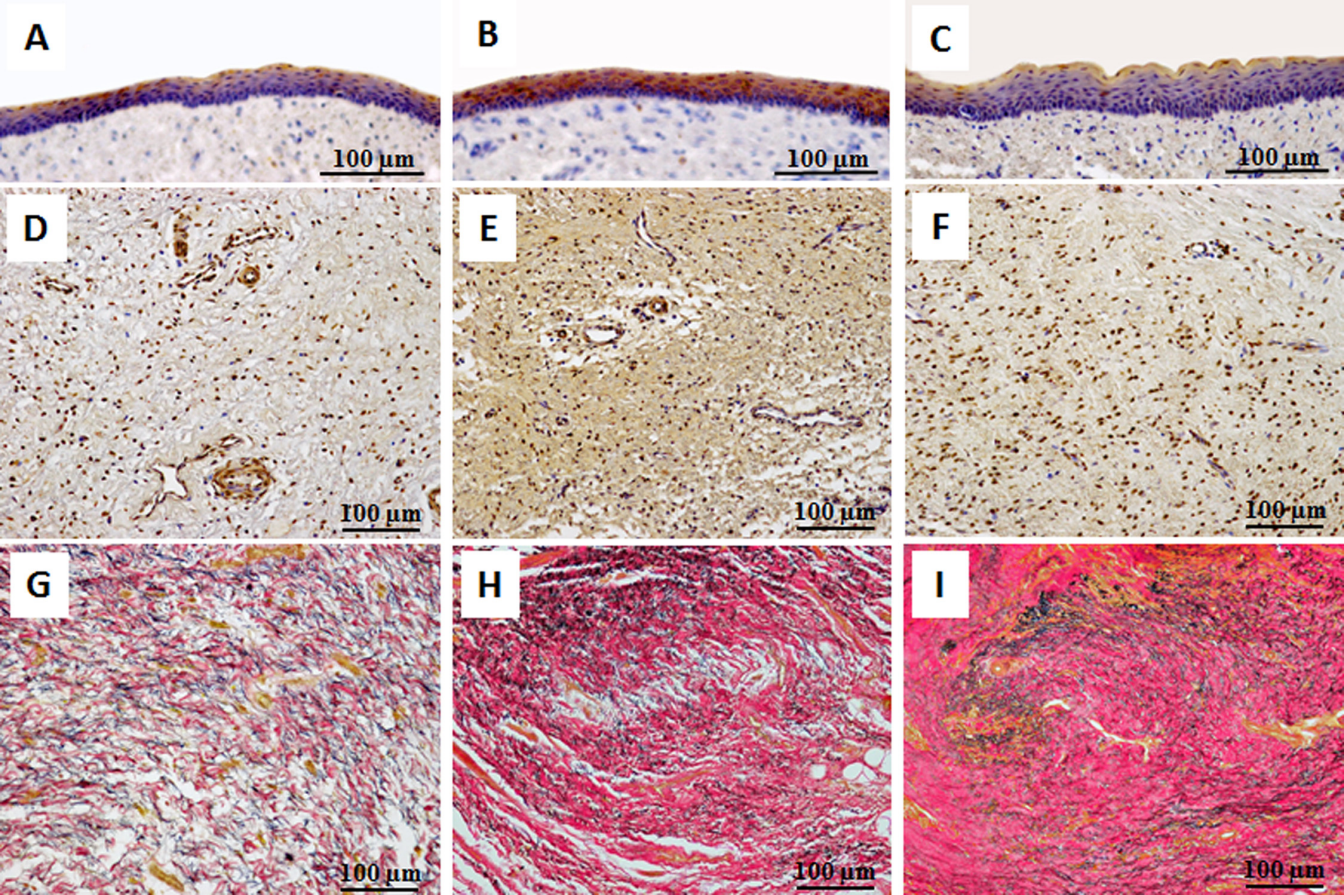
Histological comparison between normal (A, D, E), transplanted (B, E, H), and control (C, F, I) portions of the removed larynges. Epithelial cells were not immunohistochemically stained with anti-cytokeratin 8 antibodies in normal (A) or control (C) portions, but stained positively on transplanted portions (B). The stratified epithelium on both normal and transplanted portions consisted of five to seven uniform cell layers, but differed from the control portion, which consisted of approximately ten uniform cell layers. Cells stained with vimentin, including fibroblasts, were identified in equal density in the lamina propria on every portion (D, E, F). Vascular endothelial cells stained with vimentin were identified in greater number in the lamina propria on normal (D) and regenerated (E) portions than on control (F) portions. EVG staining revealed both collagenous and elastic fibers on transplanted portions (H), with denser collagenous fibers than those on normal portions (G). Scar tissue formation and proliferation of collagenous fibers were observed on control portions (I).

Epithelial cells did not stain positively with anti-cytokeratin 8 antibody on normal or control portions (Fig 10A and 10C), but stained positively on transplanted portions (Fig 10B). The stratified epithelium on both normal and transplanted portions consisted of five to seven uniform cell layers, but differed from control portions, which had approximately ten non-uniform cell layers. Cells stained with vimentin, including fibroblasts, were found at equal density in the lamina propria on all portions (Fig 10D, 10E and 10F). Vascular endothelial cells stained with vimentin were identified in greater numbers in the lamina propria on both normal and regenerated portions than on control portions. EVG staining revealed both collagenous and elastic fibers on the transplanted portions (Fig 10H), however collagenous fibers were denser than those on normal portions (Fig 10G). Scar tissue formation and proliferation of collagenous fibers were observed on control portions (Fig 10I).

## Discussion

The morphological and functional characteristics of the tissue-engineered layered mucosa (organotypic culture) closely resembled those of the normal vocal fold epithelium and lamina propria. Moreover, our results suggest that organotypic tissues fabricated from autologous oral mucosa could serve as effective substitutes for allografts in the reconstruction of optimally layered vocal folds. This reconstruction technique could offer substantial clinical advantages over allogeneic transplantation for treating intractable diseases such as scarring of the vocal folds.

The promotion of vocal fold re-epithelialization has the potential to prevent scarring of vocal folds, particularly when the scarred tissue is resected. In this study, in transplantation cases, the epithelium on resected portions that stained positively with anti-cytokeratin 8 antibody was derived from buccal mucosal cells and were uniformly observed, whereas in resection alone cases, resected portions of the vocal fold were covered by hyperplastic and non-uniform stratified epithelium, which were clearly delineated from surrounding tissues. This observation is consistent with a previous report [18]. Although oral epithelial cells were cultured with two to four cell layers on collagen gel containing fibroblasts *in vitro*, more layers of epithelium developed after transplantation. Generally, the re-epithelialization of a wound involves the migration of keratinocytes from the edges of the wound. During this process, keratinocyte migration and proliferation depend on the interaction of keratinocytes with subepithelial fibroblasts and the extracellular matrix (ECM) [19]. This interaction stabilizes the morphological structure and function in wound healing [20]. Therefore, the organotypic vocal fold with cultured epithelial cells can approximate an optimal epithelial layer depending on the microenvironment, creating a physical barrier that protects the compromised tissue of the subepithelium immediately after transplantation.

The characteristic morphological difference between regenerated vocal fold mucosa and controls in case of resection alone was scar tissue formation in the lamina propria after wound healing. Cutaneous wound healing has been well documented, and data pertaining to vocal fold scarring also exists. A histologic study of human scarred vocal folds reported that excessive and disorganized collagen deposition was observed in most cases that had undergone deep resection of the lamina propria, whereas collagen deposition was mild and well organized after superficial resection [21]. Another study on acute vocal fold injury reported that the vocal fold wound healing process was analogous to wound repair in the skin during the inflammatory and proliferative phases, but differed during the remodeling phase, the final phase of wound healing [22]. In the remodeling phase, the wound undergoes contraction resulting in a smaller amount of apparent scar tissue [23]. In the present study, the minor scar tissue formation in the lamina propria compared with controls two months after transplantation (i.e., in the remodeling phase) may reflect accelerated would healing in the regenerated vocal fold mucosa.

In this *in vivo* model of wound healing with transplanted tissues, the regenerated mucosa was morphologically similar to the normal one. However, the number of elastic fibers in the lamina propria was relatively low in the regenerated vocal fold mucosa. The viscoelastic properties of the human vocal fold depend on the cover, which is composed of epithelium and the superficial layer of the lamina propria. The vocal fold epithelium has a regular alignment of basal cells and multilayered squamous cells. Human ECMs of the superficial layer of the lamina propria are comprised mainly of collagen, elastin, fibronectin, and hyaluronic acids [18, 24, 25]. These ECMs provide mechanical strength and resistance to shear stress. The main features of vocal fold scarring are disorganized collagen and elastin bundles, loss of important ECM constituents, volume deficiency, loss of vocal fold pliability, and glottal insufficiency [26, 27]. For optimal regeneration of the vocal folds, further adjustments to the ECMs in the lamina propria are needed.

In this study, the regenerated vocal fold mucosa, as well as the fabricated organotypic cultured tissues, consisted of uniform epithelium in the upper layers and collagen, including fibroblasts, in the lower layers (the lamina propria). Since the fibroblast direction was aligned with the oriented collagen sheet [28, 29], these cells could develop in the lamina propria while maintaining anterior to posterior binding strength as a vibrating tissue. These oriented ECMs are the most important structures for vocal fold mucosal waves [18, 24, 25].

Our stroboscopic examination revealed severe scar formation two months after type II cordectomy in canine models. Laryngostroboscopy can provide a slow motion-like view of vocal fold vibration (mucosal wave) by using a synchronized strobe light. It is the key tool used to analyze vocal fold vibration after cordectomy, which is a common cause of iatrogenic vocal fold scarring. In a retrospective analysis of patients who underwent laser cordectomy for early glottic carcinomas, postoperative amplitude and mucosal wave patterns were reduced proportionally to the amount of removed cordal tissue [30]. Another stroboscopic study showed that larger glottal gaps, scarring, and decreased mucosal waves were more frequently observed in cordectomies of type III-V (transmuscular to extended) compared to type I (subepithelial) or type II (subligamental) [31]. On the other hand, Kishimoto et al. reported that, while there were individual variations in temporal changes of mucosal wave amplitude and glottal gap, both parameters appeared to stabilize about 6 months after cordectomy [32]. Thus, stroboscopic abnormalities are commonly associated not only with the depth of resection, but also the postoperative period or severity of scar formation after cordectomy.

One technique that has been shown to restore vocal fold scarring is the use of free buccal mucosa grafts. Isshiki [33] has used this type of direct transplantation in human subjects with an open technique and rubber stent fixation via a laryngofissure. However, our strategy has several advantages over the free buccal mucosa graft. First, a smaller piece of mucosal tissue is initially harvested. As a result, wound healing was facilitated within several days without incident or scarring. Second, the organotypic cultured tissue is of a more uniform thickness, and is morphologically distinct from free buccal mucosa grafts, which are much thicker, multilayered, and have irregular surfaces. Third, the organotypic cultured tissues promote faster regeneration through interactions between the epithelium and fibroblasts in the three-dimensional tissue reconstruction, as stated above. These tissues not only accelerate proliferation of epithelial basal cells, but also inhibit the growth of fibrous tissue [34]. In the future, organotypic cultured tissues with tissue-engineered structures may enable us to create a new vibrating structure.

There are some limitations and ethical issues that must be resolved before this technique can be applied in human clinical trials. First, the transplantable cultured cells in this study used the common 3T3 feeder layer method, which was originally developed for the production of autologous mucosal grafts. This method has been clinically applied since the 1980s for the treatment of various skin conditions, including burns and giant nevi, although the U.S. Food and Drug Administration classifies these grafts as xenografts. A research model for organotypic vocal fold mucosa should be made without using the 3T3 feeder-layer method [35]. Second, the canines frequently used in laryngeal research [36] are not perfect animal models for all voice research due to their lack of vocal ligaments. Third, the loss of cellular adhesion in the cultured epithelial autograft is associated with epithelial-mesenchymal transition and tumor progression in epithelial carcinomas. Finally, long-term follow-up and experimentation on a large number of animal models are necessary to further assess the benefits and risks of this method, which offers the potential to treat vocal fold scarring that is resistant to standard approaches. We believe that modification of our technique will lead to successful clinical application of organotypic vocal fold mucosa preparations in the future.

## Conclusion

This study experimentally fabricated tissue-engineered vocal fold mucosa from canine oral mucosal cells. Using this organotypic cultured mucosa with tissue-engineered autologous oral mucosa, the vocal fold mucosa was successfully restored. Our results suggest that this reconstruction technique could offer substantial clinical advantages for treating intractable diseases such as scarring of the vocal folds.
